# Type V tentorial dural arteriovenous fistula: a case of augmented reality–assisted surgical salvage

**DOI:** 10.3171/2025.7.FOCVID2541

**Published:** 2025-10-01

**Authors:** Chih-Wei Huang, Chung-Hsin Lee, Kai-Chen Chung, Wen-Hsien Chen, Yuang-Seng Tsuei

**Affiliations:** 1 Institute of Medicine, Chung Shan Medical University, Taichung;; 2 Department of Neurosurgery, Neurological Institute, and; 3 Department of Radiology, Taichung Veterans General Hospital, Taichung; and; 4 Department of Post-Baccalaureate Medicine, College of Medicine, National Chung Hsing University, Taichung, Taiwan

**Keywords:** tentorial dural arteriovenous fistula, augmented reality–assisted microsurgery, endovascular embolization, CISS MRI, Cognard classification

## Abstract

This video demonstrates the case of a Cognard type V tentorial dural arteriovenous fistula that was successfully salvaged using augmented reality–assisted microsurgery following failed endovascular embolization. The patient had a spastic gait and quadrilateral numbness. MRI showed medullary edema and abnormal vessels near the tentorium. DSA confirmed a shunt fed by the meningohypophyseal trunk draining into perimedullary veins. Initial embolization was unsuccessful. Using CISS MRI and augmented reality projection, the authors identified and ligated the arterialized veins, relieving venous hypertension. Postoperative imaging showed improvement of the brainstem edema.

The video can be found here: https://stream.cadmore.media/r10.3171/2025.7.FOCVID2541

## Transcript

### 0:26 Case Presentation.

This is a case of a middle-aged man who came in with numbness in all four limbs and difficulty walking for about 3 months. On neurological examination, he had clonus and hyperreflexia in both lower extremities—classic signs pointing toward an upper motor neuron lesion.

### 0:47 Imaging Findings.

An initial MRA was performed at an outside hospital. The preoperative MRI showed noticeable edema in the medulla oblongata, along with abnormally engorged vessels in the posterior fossa. These findings raised a strong suspicion of abnormal vascular shunting leading to brainstem congestion. As a result, the patient was referred for a diagnostic cerebral angiogram.

### 1:17 First DSA Findings.

A digital subtraction angiography was performed, starting with the left internal carotid artery. In the arterial phase, we observed early venous filling—an abnormal finding. The feeding artery was identified as the medial tentorial artery, which originates from the meningohypophyseal trunk. Arteriovenous shunting was noted at the level of the tentorium, with venous drainage extending down into the posterior fossa and cervical spinal veins. This DAVF was classified as Cognard type V^1–5^ due to its perimedullary venous drainage without dural sinus involvement. The shunting occurred at the tentorium, with venous reflux descending toward the spinal perimedullary veins, causing spinal cord ischemia due to venous hypertension.

### 2:09 Treatment Planning.

This case featured a single main arterial feeder and one draining vein. Treatment options include: plan A, transarterial or transvenous embolization; and plan B, surgical disconnection to interrupt the fistulous point and restore normal venous outflow.

### 2:28 Plan A—Transarterial Embolization.

Our initial plan was transarterial embolization through the meningohypophyseal trunk. However, during the embolization, Onyx glue unexpectedly refluxed into the ICA, which led to the termination of the procedure. Fortunately, the interventional neuroradiologist was able to retrieve the Onyx glue without any treatment-related complications. Follow-up DSA showed that the DAVF structure remained unchanged due to incomplete embolization.

### 3:02 Plan B—Craniotomy.

Since the transarterial approach failed, we proceeded with plan B—surgical disconnection of the draining vein through a craniotomy.

### 3:12 Surgical Approach.

The patient was positioned in the park-bench position, and a left retrosigmoid craniotomy was performed. Traditionally, arterialized draining veins are identified by their thickened walls and high-pressure flow, as seen here.

### 3:35 Preoperative Planning With CISS MRI.

Due to the complexity and variability of the draining vein anatomy in the posterior fossa, we used CISS MRI^6^ to enhance the visualization of cranial nerves and vascular structures by taking advantage of CSF contrast. Using CISS MRI data, we segmented and reconstructed 3D models of the trigeminal, facial, and vestibulocochlear nerves, as well as the abnormal drainage vein. These models were imported into the navigation system.

### 4:10 Intraoperative Imaging.

Navigation was used intraoperatively to integrate the presegmented 3D models. This allowed us to correlate real-time anatomy with preoperative imaging. Based on angiography and 3D reconstruction, a Y-shaped bifurcation was identified, suggesting either a branch or convergence of veins. The caudal limb extended toward the cervical spine. In this case, we projected the segmented facial nerve, vestibulocochlear nerve, trigeminal nerve, and the draining vein onto the microscope view using AR.^7,8^ This enabled intuitive localization and disconnection of the target vein without the need for mental image-spatial conversion.

### 4:56 AR Versus Traditional View.

On the left is the traditional microscopic view of the posterior fossa. On the right, AR overlay shows green for the facial nerve, yellow for the trigeminal nerve, and purple for the two arterialized veins. We successfully interrupted both.

### 5:12 Surgical Ligation.

After we carefully dissected and identified the cranial nerves and the abnormal shunting vessels, the vascular structures could be easily distinguished with the help of AR projection.^7,8^

Following thorough coagulation, we disconnected the abnormal vessel connection, successfully relieving the abnormal venous reflux and venous hypertension.

### 5:40 Pre- and Postoperative Comparison.

This is a comparison image before and after surgery. ICG fluorescence imaging allows us to clearly visualize the vascular anatomy before ligation and shows the disappearance of the abnormal shunting vessels postoperatively, while confirming preservation of the normal vasculature. One month after surgery, follow-up MRI showed resolution of the medullary edema, indicating successful reduction of the venous hypertension of the brainstem.

## Disclosures

The authors report no conflict of interest concerning the materials or methods used in this study or the findings specified in this publication.

## Author Contributions

Primary surgeon: Tsuei, Huang. Assistant surgeon: Lee. Editing and drafting the video and abstract: Huang, Chung. Critically revising the work: Tsuei, Huang, Chung. Reviewed submitted version of the work: Huang, Chung, Chen. Approved the final version of the work on behalf of all authors: Tsuei. Supervision: Chen.

## Supplemental Information

### Patient Informed Consent

The necessary patient informed consent was obtained in this study.

## References

[1] Cannizzaro D Brinjikji W Rammos S Murad M Lanzino G Changing clinical and therapeutic trends in tentorial dural arteriovenous fistulas: a systematic review AJNR Am J Neuroradiol 2015 36 10 1905 1911 26316563 10.3174/ajnr.A4394 PMC7965046

[2] Cognard C Gobin YP Pierot L Cerebral dural arteriovenous fistulas: clinical and angiographic correlation with a revised classification of venous drainage Radiology 1995 194 3 671 680 7862961 10.1148/radiology.194.3.7862961

[3] Gross BA Cerebral dural arteriovenous fistulas Stroke Vasc Interv Neurol 2022 2 4 e000532 10.1161/SVIN.122.000532 PMC12778802 41584768

[4] Lawton MT Sanchez-Mejia RO Pham D Tan J Halbach VV Tentorial dural arteriovenous fistulae: operative strategies and microsurgical results for six types Neurosurgery 2008 62 3 Suppl 1 110 125 18424975 10.1227/01.neu.0000317381.68561.b0

[5] Satomi J van Dijk JMC Terbrugge KG Willinsky RA Wallace MC Benign cranial dural arteriovenous fistulas: outcome of conservative management based on the natural history of the lesion J Neurosurg 2002 97 4 767 770 12405361 10.3171/jns.2002.97.4.0767

[6] Blitz AM Macedo LL Chonka ZD High-resolution CISS MR imaging with and without contrast for evaluation of the upper cranial nerves: segmental anatomy and selected pathologic conditions of the cisternal through extraforaminal segments Neuroimaging Clin N Am 2014 24 1 17 34 24210310 10.1016/j.nic.2013.03.021

[7] Huang CW Lee CH Chung KC Tsuei YS Feasibility and workflow analysis of IV-DSA-based augmented reality-guided brain arteriovenous malformation resection in a hybrid operating room: i-Flow tailored method J Neurointerv Surg 2025 17 3 332 37770182 10.1136/jnis-2023-020797 PMC12163979

[8] López WOC Navarro PA Crispin S Intraoperative clinical application of augmented reality in neurosurgery: a systematic review Clin Neurol Neurosurg 2019 177 6 11 30579049 10.1016/j.clineuro.2018.11.018

